# Comparative effectiveness of videotape and handout mode of instructions for teaching exercises: skill retention in normal children

**DOI:** 10.1186/1546-0096-10-4

**Published:** 2012-01-30

**Authors:** Garima Gupta, Stuti Sehgal

**Affiliations:** 1 Lecturer, M.P.T Neurology, Saaii College of Medical Science and Technology, Kanpur, India; 2 Lecturer, M.P.T Neurology, ISIC Institute of Health and Rehabilitation Sciences, New Delhi, India

**Keywords:** Physical therapy, motor skill, mode of instructions, patient education material, learning strategies, readability, instructional principles

## Abstract

**Background:**

Teaching of motor skills is fundamental to physical therapy practice. In order to optimize the benefits of these teaching and training efforts, various forms of patient education material are developed and handed out to patients. One very important fact has been overlooked. While comparative effectiveness of various modes of instruction has been studied in adults, attention has not been paid to the fact that learning capabilities of children are different from that of adults. The intent of the present study is to compare the effectiveness of video and handout mode of instructions specifically on children.

**Methods:**

A total of 115 normal elementary-age children aged 10 to 12 years of age were studied. The children were randomized into two groups: A) the video group, and B) the handout group. The video group viewed the video for physical therapy exercises while the handout group was provided with paper handouts especially designed according to the readability of their age group.

**Results:**

Statistical analysis using the student's't' test showed that subjects of both the video and handout groups exhibited equal overall performance accuracy. There was no significant difference between the groups both in acquisition and retention accuracy tests.

**Conclusion:**

The findings of the present study suggest that if the readability and instructional principles applicable to different target age groups are strictly adhered to, then both video as well as handout modes of instructions result in similar feedback and memory recall in ten to twelve year-old children. Principles of readability related to the patient age are of utmost importance when designing the patient education material. These findings suggest that the less expensive handouts can be an effective instructional aid for teaching exercises to children with various neuromuscular, rheumatic, and orthopedics conditions and the most costly videotape techniques are not necessarily better.

## Background

The theoretical model of motor learning has three aspects: 1) cognitive processes; 2) motor command; and 3) sensory feedback. Cognitive processes comprise the collective group of thoughts which help the learner in decision-making process regarding the anticipated planning, regulation and interpretation of motor performance [1]. Knowledge of these learning models is crucial to prescribing the relevant exercises to patients. Then exercise prescription is fundamental to physical therapy (PT). This prescription starts with the child and parents learning the prescribed exercises correctly and then hopefully remaining adherent to them at home. This two-step process is the key to success of such a physical therapy treatment program.

Previous research has shown that 65% of patients are non-adherent to some degree to a PT program, for example, they are not fully adherent to the instructions [2]. No doubt there are multiple factors which may affect patient and family adherence to performance of the home treatment program, but learning the exercises appears to be of prime importance. Learning the exercises may be best done by "observational learning" which is the application of demonstration [3]. The principal theoretical influence of this model is to leave the learner, a child or family member, with a conception of the way a skill is to be performed. In this way, the learner is spared from the extra burden of creating a cognitive conception of the desired action pattern and thus may improve the efficiency of skill acquisition [4].

Learners appear to code this modeling as a symbolic behavior which becomes the "perceptual blue print" and serves both as a representation of what is to be done and as a referent for making corrections [1]. Interestingly, positron emission tomography (PET) brain scanning suggest that there is considerable overlap in the cortical areas of the brain that are activated when human beings perform an action and also when they view another individual performing the action [5,6].

Physical therapists use various modes of instructions such as videotape, auditory, paper handouts, tactile, cognitive strategies, rehearsal, or live one-on-one personalized instruction [7-10]. All these different types of instructions serve as an external reference for performance. There are limited studies that compare the effectiveness of video and handout mode of instructions. Friedrich studied the effect of the simple use of a brochure being given out versus instructions by the physical therapist with patients who were experiencing pain in the neck and lower back region. The study revealed that whatever is been directly communicated is more effective than giving out a brochure without oral instructions [11]. In this study it was not clear whether the better performance of the supervised group of patients was due to the quality of the dynamic direct mode of instruction by each therapist. Another study compared the videotape mode of instruction versus illustrations for influencing quality of performance on healthy adults. Findings of this study suggested that instruction via videotape was more effective than static illustrations. The weakness of this study may be that it was not clear how readable and understandable the illustrations were. Therefore, it is not clear that a poor performance of the illustration group was due to unclear instructions or from the static mode of instruction. These studies mainly focus on the elderly or young adult population.

Children use feedback in a manner significantly different from that of adults [12,13]. Cognitive processes such as selective attention and speed of information processing increases with age. Children use different strategies than adults to process information in tasks that require visual-spatial working memory, object-recognition memory, verbal learning, copying spatial patterns, or higher-level attention focusing [13]. Attention focusing is the act of directing attention to information sources or to objects placed in front of an individual.

Therefore teaching exercises to the children is a challenging task. There are various conditions in which physical therapists teach exercises to children suffering from cerebral palsy, polio, fractures, rheumatoid arthritis, and other diseases. But there is no published literature which says which specific mode of instruction will result in better learning in children. The intent of this study is to compare the effect of two different forms of instructional aides used to improve the performance accuracy of the physical therapy learning exercise. The two forms of instructions that are compared in this study are the videotape and display paper handout for the same set of exercises. As per previous findings, we hypothesized that the performance accuracy of the subjects who viewed the videotape is higher than the group that was given the display paper handout. The Null hypothesis is that in the case of skill retention of taught exercises in normal children, there is no difference between videotape and handout modes of instructions.

## Methods

The study was approved by external research committee of Guru Gobind Singh Indraprasth University, New Delhi. A total of 115 volunteers in the 10-12 year age-group from one of the secondary schools in New Delhi were recruited. A signed consent form was obtained from the guardians of the subjects. While selecting the volunteers all the subjects were assessed on "mini mental status examination" (MMSE) and asked for hand dominance.

Normal secondary school children of the age group 10 to 12 years were included if they had no recent upper limb injury which can affect upper limb full range of motion or cause pain in movement. The modified MMSE cutoff score was set at 30 for 10 to 11 year old children and 35 for 12 year old children [14]. Only normal children with corrected vision and hearing who could understand English were considered.

Children who had known neurological, musculoskeletal, cardiovascular or psychiatric disorder were excluded. Children who could not follow the instructions were also excluded from the study.

**Figure 1:**
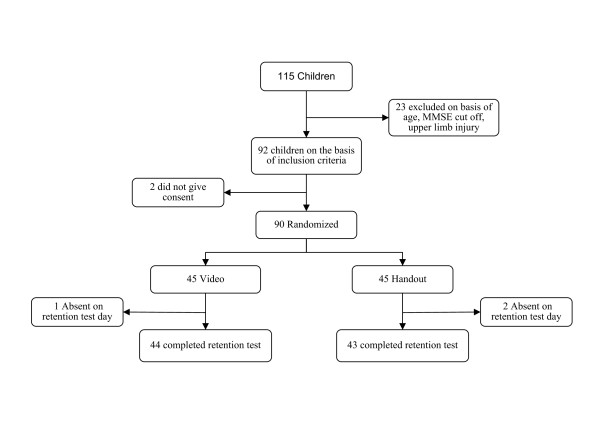


The subjects who fulfilled the inclusion criteria were then selected and randomly assigned to either the video group (A) or the handout group (B), using random number tables (Figure 1). Demographic details (name, age, gender) of the subjects were collected and assessment was done (Table 1). The protocol consisted of an acquisition test on day one. In this the subject viewed the exercises either with video or a handout. The test conducted at the end of session is called the acquisition test. This test measures the immediate performance of the subject but it may or may not reveal the amount of learning. On day two retention tests was conducted, following a one day break from practice. In this retention test conditions remain the same as in the acquisition test. This test measures the amount of retained information in terms of learning. There were five exercises for both the groups. Each subject performed the exercises in the standing position with a yellow Thera-Band:

**Table 1:** Components used for scoring each exercise with number of errors committed

Exercise	Exercise Scoring Components	Number of errors					
		**Video Group**			**Handout Group**		
		
		**Day1**	**Day2**	**Total**	**Day1**	**Day2**	**Total**
		
1. Both arms "W" shoulder blade squeeze	Hands in front less than shoulder width apart	7	3	10	20	14	34
	
	Elbows bent the same angle throughout exercise	10	7	17	11	6	17
	
	Scapular retraction (elbows extend beyond frontal plane)	9	9	18	3	6	9
	
	Band snug without slack or snapping	7	0	7	0	0	0

2. One arm elbow straightening	Both arms begin with elbows bent and hands at respective shoulders	26	11	37	51	48	99
	
	Anchor arm remains bent in same position	31	24	55	27	28	55
	
	Dominant arm extends to side (< 45° of abduction)	10	14	24	15	16	31
	
	Band snug without slack or snapping	0	0	0	3	3	6

3. One arm "full" soda can exercise	Thumb begins and remains pointed up	24	25	49	38	33	71
	
	Arm raised only to shoulder level (< 120° of flexion)	26	34	60	21	22	43
	
	Elbow begins and remains straight	24	21	45	15	16	31
	
	Band under one foot without slack	3	3	6	0	1	1

4. "Shoulder clocks"	Elbows remain straight the entire time	30	29	59	6	9	15
	
	Rotation occurs only at the shoulders	28	20	48	9	9	18
	
	Rotation occurs, beginning forward	24	20	44	19	30	49
	
	Band is secured under both feet without slack or snapping	3	8	11	0	6	6

5. Double arm "V" exercise	Elbows begin and remain straight the whole time	1	1	2	3	3	6
	
	Arms raise together overhead to "V" position	7	10	17	0	0	0
	
	Arms in scapular plane	4	4	8	3	3	6
	
	Thumbs pointed up the whole time	3	3	6	3	3	6

TOTAL ERRORS		277	246	523	247	256	503

(1) bilateral scapular retraction or "both arms 'W' shoulder blade squeeze";

(2) unilateral elbow extension or "one arm elbow straightening";

(3) unilateral shoulder flexion in scapular plane or "one arm 'full' soda can exercise";

(4) bilateral shoulder circles or "shoulder clocks"; and

(5) bilateral shoulder flexion or "double arm 'V' exercise" (performed without the resistive band) (Table 1).

Performance assessment of subjects was done on a score sheet specifically designed for those particular exercises. Julie et al. have previously assessed the reliability of this score sheet with an intra and inter class correlation coefficient at 0.98 and 0.95, respectively [9]. The scoring sheet had four critical components for each exercise, which provided the basis for scoring and analysis of exercise performance.

These critical components were based on elements that are often performed incorrectly by patients but are deemed essential for correct completion of each exercise. The score attained by the subjects were accordingly recorded.

In group 'A' demonstration of all five exercises were given through videotapes. The video included the modeling along with the verbal instructions In the video, the researcher was the model and the personal SONY 15x optical zoom camera was used. The instructions given in the video were similar to that of handout group 'B', which received the handout of each exercise on a separate A4 size sheet. The handout consisted of five pages depicting the starting and the end position of each exercise along with the written instructions below each exercise. The readability of written instructions was assessed by 'Flesch Kincaid Reading Ease' and 'Flesch Kincaid Grade Level' formulas [15-17].

In the school premises, a separate quiet room was chosen for conducting the tests. Initial instructions about the whole procedure were provided in age-appropriate simple and clear words and then no additional questions or conversation was allowed. The same time of the day was used during the entire study. All subjects wore a short sleeved shirt to allow clear visualization of the elbow and arm for scoring. Each of the group 'A' subjects viewed the video alone in a room and was instructed not to practice along with The video; similarly, each of the group 'B' subjects was provided with handouts alone in a room and was instructed not to practice as each read the handout. The video consisted of a total of seven demonstrations of each exercise. The time allotted to read the handout was same as of the given video of the exercise. After this, five consecutive practice trials were given for each exercise and thereafter the subject was provided with the thera band. The immediate acquisition test on day one was done ten minutes after the demonstrations and the data was recorded on the score sheet [18].

For the retention tests on day two, no video or handout was shown to the subjects. They were asked to recall all the five exercises and perform each of them. Again data was recorded on the scoring sheet. The subjects were allowed the liberty to perform the exercises in any order, completing five repetitions of each exercise. As mentioned earlier, each exercise had four components. For scoring, the subject received one point for each correct exercise component performed. The maximum score was twenty for the series of five exercises. Subjects were asked to perform each exercise five times and only the middle three repetitions were considered for scoring. With these three repetitions of each exercise maximum total score of each subject was sixty. The score was then finally analyzed using statistical tools SPSS software version 17.0. A student's t- test was used to analyze the difference between the performance accuracy scores of both the groups for both the acquisition phase and the retention phase. For all statistical tests the level of significance was set at P ≤ 0.05.

## Results

**Figure 2:**
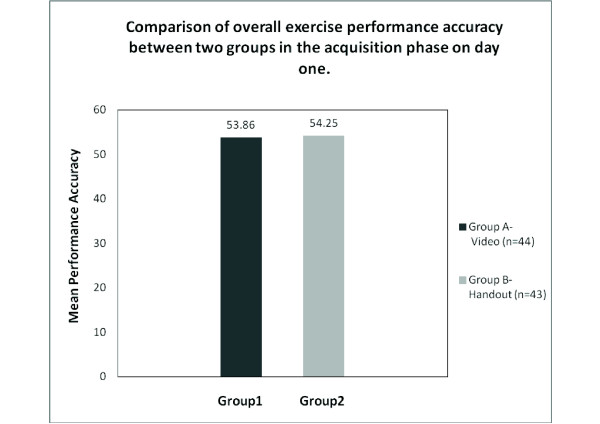


**Figure 3:**
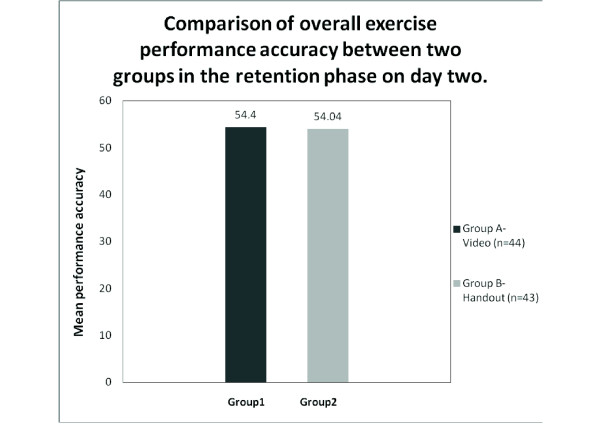


The group 'A', which viewed the video comprised of 25 males and 19 females with a Mean ± SD age of 10.75 ± 0.84 years. Group B, which viewed the handouts comprised of 32 males and 11 females with a Mean ± SD age of 10.83 ± 0.84 years (Table 2). The statistical analysis using unpaired 't' test on acquisition day between the groups showed no significant difference (Figure 2). The 't' value for the acquisition phase was 0.27 (p value ≤ 0.79) (Table 3). The analysis of retention test also does not show any significant difference between the groups (Figure 3). The 't' value for the retention phase was 0.29 (p value ≤ 0.77) (Table 3).

**Table 2:** Basic characteristic of subjects

Characteristic	Subjects
Total number of subjects	87

Male and Females	Group A- 25 males and 19 females
	
	Group B- 32 males and 11 females

Mean age years ± SD	Group A- 10.75 ± 0.84
	
	Group B- 10.83 ± 0.84

**Table 3:** Comparison of overall exercise performance accuracy between the two groups in the acquisition phase on day one and on the retention phase day two

Days	Group1 (N = 44)		Group 2 (N = 43)		't' value	P value
			
	Mean	SD	Mean	SD		
Acquisition phase GTD1	53.86	7.12	54.25	6.20	0.27 ^NS^	0.79

Retention phase GTD2	54.4	5.71	54.04	5.86	0.29 ^NS^	0.77

GTD1: grand total day one, GTD2: grand total day two, NS: non significant

## Discussion

Currently videotapes are widely used as education material in physical therapy and are very much in vogue. Yet videotapes are expensive and out-of-reach of low income group of patients in remote areas. There is a definite need to question whether the inexpensive and readily available traditional handout alternative to the videotape can be utilized in educating patients and families and be just as effective.

The key finding of this study is that the instructions conveyed to subjects via videotapes and via handouts exhibited equal overall performance accuracy and both the groups closely matched the conception of the exercises, thereby resulting in similar memory recall and providing relevant attention-focusing information on the exercises.

The results of this study are in line with the result of Meade et al. findings in adults with colon cancer that also indicated an equal effectiveness between printed handout and videotaped material. Meade et al. suggested that their findings might be ascribed to: 1) The interventions were tailored to the target group, with special attention given to developing content relevant to their learning needs; 2) organizing content in a clear manner; 3) using an active voice; 4) writing or narrating in a conversational style; 5) using short words and sentences; and 6) the usage of specialized pretesting tools [19].

In the present study, both video and handout instructions were developed in accordance with similar principles. The video was modeled by the researcher and the verbal instructions were given in a clear and active narrative voice [1]. The handouts were augmented with written instructions in simple, clear, and short sentences. Key information was emphasized by letters in a bold font.

Readability is the ease of understanding due to the style of writing. Readability formulas are mathematical in nature. The most common factors calculated by these formulas are number of words in a sentence and number of letters or syllables per word. Most of the readability formulas calculate the difficulty of words and the difficulty of sentences. The Flesch Reading Ease formula is one of the most widely used formulas. The higher its value, the easier it is to read the text. Pre-testing of the handout material was done ensuring that the material was appropriate for reading at secondary school grades 4-5 and with a high score on the Flesch Reading Ease of 96.8. Overall, complex words were used in the entire written material at a level of only 5%. Our findings suggest that handouts that are written at high readability levels and videotapes that contain concrete, simple, active, narrative instructions would be acceptable and more appropriate for our target population in India and other developing countries.

The performance accuracy of both the video and handout groups was high and closely matched each other. This high accuracy and matching results could be due to the special focus and extra effort given to development of the instructions material.

Available literature on motor learning in children suggests that if the focus of attention is directed internally instead of externally, it should result in better learning. An internal focus of attention in this case means attention on the whole movement or action that provides increased enhancement of motor learning and performance [20]. The instructions given in the present study were intended to provide information about the whole movement pattern as well as its subdivisions, which helped the children in using their attention focusing ability more efficiently. For example in exercise 3 the subject was instructed to pull the band keeping the thumb towards the ceiling (internal focus of attention) whereas if the same instruction was meant for an adult he would be asked to pull the band upwards so that the thumb is at right angle to the ground (external focus of attention).

The target age group of subjects in the present study is in the transition stage of developing and generalizing on their cognitive skills [21,22]. This could possibly explain the errors committed by the children in positioning their limbs in most of the exercises as there was no visual feedback provided to them. Similarly, in a series of studies that compared situations in which the subjects were able to monitor the performance visually or not reported the use of concurrent visual monitoring had a significant positive effect on immediate and long term accuracy of motor skill performance [17,23].

Further studies are needed to examine the effectiveness of both video and handout modes of instructions with children of various age groups and genders, as well as the various neurological and orthopedics conditions, on the comparative long time adherence and motivation using the observational learning strategies. Future studies on the effects of different mode of instructions for complex motor skills are also needed.

One limitation of this study was that although normal subjects were utilized for the study, there was no any previous study of a similar nature which could offer the comparative data on how children use the video and handout modes of instructions; conversely, this limitation may also be a strength as this study may provide novel insights into the learning process of children. Other limitations were that long time retention was not tested and blinding was not done. Also, the motivation level of the learner was assessed subjectively.

## Conclusions

The findings of this present study suggest that if the readability and instructional principles are followed while giving both video and handout modes of instructions to the target age group, it will result in similar feedback and memory recall on skill retention of taught exercises in ten to twelve year old children. Thus the experimental hypothesis was rejected and null hypothesis was accepted.

There are some practical implications of this work. Physical therapists prescribe exercises usually to correct specific muscle or muscle-group performance and to minimize injury. If an exercise is performed incorrectly, then desired recovery in performance of the targeted muscle or muscle-group is less likely to occur. This result is clearly highlighted through the modeling presented in this study. Along with motor skill teaching, it is equally essential to ensure that the learner is able to perform the exercises without any supervision. This study thus emphasizes the importance of mode of instructions in the process of motor learning by showing how children use both video and handout modes of instructions. This understanding can be used as an important tool for designing a home-based unsupervised treatment protocol, for development of patient education material for young children for physical development, and for training of patients with neurological or musculoskeletal problems. When working with these children, it is crucial that the physical therapist keep the instructional feedback simple and more narrative to provide optimal learning.

## Conflict of interest

All the authors read and declare that we have no competing interests.
